# From Strain Stiffening to Softening—Rheological Characterization of Keratins 8 and 18 Networks Crosslinked via Electron Irradiation

**DOI:** 10.3390/polym14030614

**Published:** 2022-02-04

**Authors:** Iman Elbalasy, Nils Wilharm, Erik Herchenhahn, Robert Konieczny, Stefan G. Mayr, Jörg Schnauß

**Affiliations:** 1 Peter-Debye Institute for Soft Matter Physics, Leipzig University, Linnéstraße 5, 04103 Leipzig, Germany; eh12veli@studserv.uni-leipzig.de; 2 Faculty of Science, Cairo University, Giza 12613, Egypt; 3 Leibniz-Institut für Oberflächenmodifizierung e.V. (IOM), Permoserstr. 15, 04318 Leipzig, Germany; nils.wilharm@iom-leipzig.de (N.W.); robert.konieczny@iom-leipzig.de (R.K.); 4 Division of Surface Physics, Department of Physics and Earth Sciences, Leipzig University, Linnéstraße 5, 04103 Leipzig, Germany; 5 Fraunhofer Institute for Cell Therapy and Immunology, Perlickstraße 1, 04103 Leipzig, Germany; 6 Unconventional Computing Lab, Department of Computer Science and Creative Technologies, UWE, Bristol BS16 1QY, UK

**Keywords:** k8–k18, rheology, electron beam irradiation, strain stiffening, strain softening, permanent crosslinking

## Abstract

Networks of crosslinked keratin filaments are abundant in epithelial cells and tissues, providing resilience against mechanical forces and ensuring cellular integrity. Although studies of in vitro models of reconstituted keratin networks have revealed important mechanical aspects, the mechanical properties of crosslinked keratin structures remain poorly understood. Here, we exploited the power of electron beam irradiation (EBI) to crosslink in vitro networks of soft epithelial keratins 8 and 18 (k8–k18) filaments with different irradiation doses (30 kGy, 50 kGy, 80 kGy, 100 kGy, and 150 kGy). We combined bulk shear rheology with confocal microscopy to investigate the impact of crosslinking on the mechanical and structural properties of the resultant keratin gels. We found that irradiated keratin gels display higher linear elastic modulus than the unirradiated, entangled networks at all doses tested. However, at the high doses (80 kGy, 100 kGy, and 150 kGy), we observed a remarkable drop in the elastic modulus compared to 50 kGy. Intriguingly, the irradiation drastically changed the behavior for large, nonlinear deformations. While untreated keratin networks displayed a strong strain stiffening, increasing irradiation doses shifted the system to a strain softening behavior. In agreement with the rheological behavior in the linear regime, the confocal microscopy images revealed fully isotropic networks with high percolation in 30 kGy and 50 kGy-treated keratin samples, while irradiation with 100 kGy induced the formation of thick bundles and clusters. Our results demonstrate the impact of permanent crosslinking on k8–k18 mechanics and provide new insights into the potential contribution of intracellular covalent crosslinking to the loss of mechanical resilience in some human keratin diseases. These insights will also provide inspiration for the synthesis of new keratin-based biomaterials.

## 1. Introduction

Filamentous biopolymers constituting the cytoskeleton and extracellular matrix (ECM) are the essential ingredients of life, maintaining cell shape and mechanically supporting cells and tissues. Unlike purely synthetic polymers, these biopolymers are able to actively reconfigure themselves in response to environmental conditions [1]. Therefore, they endow the cell and tissue with a paradoxical combination of mechanical strength and dynamics to withstand large mechanical loads with the ability to grow and reshape—both are crucial aspects for life. To fulfill these functions, the architecture of these filamentous proteins needs to be highly dynamic in terms of the spatial arrangement of constituting filaments as well as their connectivity [2]. The change of architecture and connectivity within networks, for instance, via crosslinking complexes, enable cells to rapidly tune their mechanical properties [3]. Simplified cytoskeletal model systems have been developed via an in vitro reconstitution approach to experimentally study the fundamental physics governing this system [4,5,6,7,8]. Keratins are crucial cytoskeletal proteins with unique material properties even at comparably low volume fractions [9]. With their 37 cytoplasmic members, they constitute the largest group of the intermediate filament (IF) proteins family within the cytoskeleton [10]. They are typically expressed in epithelial cells in various combinations and provide crucial cell-type-specific structural support and resistance against mechanical stresses. These diverse mechanical properties are realized by expressing various specific keratin pairs, as keratins obligate heterodimeric complexes representing parallel coiled coils made from two alpha-helical molecules of two sequence-related classes each [11]. Rheological studies of the in vitro reconstituted keratins showed that keratins can self-assemble into highly elastic networks with both the elastic and viscous moduli weakly depending on the deformation frequency [12,13,14,15,16]. Under large deformations, keratins display increasing stiffening even in the absence of external crosslinkers with the ability of quick recovery after breakage [15,16,17]. They show high extensibility due to their hierarchical structure and partial unfolding of subunits without filament breakage, which is considered to be the origin of the strain stiffening of IF [18,19]. From these in vitro studies, it has become clear that the unusual mechanical resilience and high extensibility of epithelial cells are mostly owing to their high keratins content, which has been supported by several cell studies [18,20,21]. In cells, the molecular mechanisms that link IFs together are not well defined, unlike the other major cytoskeletal components, which have a large number of specialized crosslinking proteins [22]. In vitro keratin networks are commonly crosslinked transiently via counterions changing their architecture and mechanics. It has been reported that adding MgCl_2_ or KCl stiffens k8–k18 networks and leads to filament bundling [12,16,23,24]. In materials science, the unique mechanical features of keratins, in addition to being a natural polymer, make them attractive candidates. Keratin-based hydrogels have attracted great interest with applications in drug delivery, wound dressings, and cell scaffolding [25,26,27]. Depending on the application, the crosslinking of fabricated gels is generally a valuable approach to modify and/or improve the material properties. One excellent technique to achieve stable chemical crosslinking is electron beam irradiation (EBI), with several advantages over traditional crosslinking methods such as glutaraldehyde. This method is reagent-free, with no potentially toxic residual reagents, making it very useful for cell studies. Additionally, it has been shown to be a convenient tool for a systematic tuning of the material properties in biopolymer networks [28,29,30,31,32]. The formation of stable covalent crosslinks between polymer chains through irradiation gives rise to a significant increase in the storage modulus of irradiated gels. The electron beam-induced hydrolysis of water molecules surrounding the polymer chains generates short-lived •OH radicals [33]. •OH radicals react with the polymer backbone to generate polymer radicals, which recombine with other macro-radicals, forming covalent crosslinks as follows:

H_2_O + ionizing radiation → •OH (1)

•OH + Polymer → H_2_O + Polymer• (2)

Polymer• + Polymer• → Polymer-Polymer(3)

A few studies have presented the modification of sheep wool and hair keratins via EBI. This irradiative procedure was found to modify the secondary structure of wool keratin, progressively increasing the β sheet over the α-helix conformation with increasing the irradiation dose and subsequently decreasing the elongation capability [34]. However, it was shown that the effect of irradiation is complex, and each dose may provoke different effects on the wool keratin structure [35]. Furthermore, several studies reported the improvement of the sorption affinity of wool keratin towards some metal cations and therefore its usefulness in environmental cleaning [36,37,38,39,40]. In a previous attempt to prepare biocompatible hair and wool keratins hydrogels via EBI, a blend with synthetic polymers was necessary to obtain a gel after irradiation [41]. Importantly, wool and hair keratins are distinguished from simple epithelial keratins (k8–k18) by their richness in cysteine amino acid, which mediates crosslinking by forming a disulphide bond, while neither k8 nor k18 contains cysteine in their structures [42].

Here, we successfully employed additive-free EBI to create covalently crosslinked k8–k18 gels. Based on this approach, we investigated the impact of permanent crosslinking on the mechanical properties of these keratin structures by treating keratin with different irradiation doses and mechanically characterizing the resultant gels via bulk shear rheology. Our study provides new insights into the mechanical behavior of crosslinked keratin structures and their contribution to cell mechanics, particularly in pathological settings.

## 2. Materials and Methods

### 2.1. Protein Preparation and Polymerization

Vectors containing keratin genes (pET 24a-k8 and pET 23a-k18), a generous gift from Harald Herrmann, DKFZ, Germany, were transformed into E. coli BL21 for protein expression. Recombinant k8 and k18 were isolated and purified as previously described [43]. For reconstitution, purified k8 and k18 proteins were mixed in equimolar amounts and renatured by stepwise dialysis against 8 M urea, 2 mM Tris–HCl, 1 mM DTT, pH 9.0 (all were purchased from Sigma-Aldrich, Germany). Each dialysis step was done for 20 min at room temperature, then the dialysis was continued overnight against 2 mM Tris–HCl, 1 mM DTT, pH 9.0 at 4 °C. The final protein concentration was determined by measuring the absorption at 280 nm with an extinction coefficient of 27,620 M^−1^ cm^−1^ for k8–k18 complex, using a DU 530 UV/vis Spectrophotometer (Beckman Coulter Inc., USA). Before irradiation, polymerization of k8–k18 was initiated by adding 1/10 volume of 10× assembly buffer (82 mM Tris-HCl, pH 7) to the protein sample to reach final conditions of 10 mM Tris-HCl, pH 7.4, and a final protein concentration of 1.5 mg/mL. The mixture of each sample (250 µL) was immediately pipetted on a glass slide and incubated and covered for one hour at room temperature for the polymerization before irradiation.

### 2.2. Electron Irradiation

The prepared samples were placed in an N_2_-filled bag to prevent side reactions between •OH and O_2_. Afterward, they were subjected to irradiation with the following doses: 30 kGy, 50 kGy, 80 kGy, 100 kGy, and 150 kGy in steps of 5 kGy to avoid sample heating. A fan further amplified the cooling. The 10 MeV linear electron accelerator (MB10-30MP; Mevex Corp., Canada) was operated with the following parameters: beam pulse duration: 15 μs, beam pulse frequency: 180 Hz. The number of pulses finally determined the dose (1 kGy = 1 J kg^−1^), whereas pulse number and dose were calibrated using a graphite dosimeter.

### 2.3. Shear Rheology

Rheology measurements were conducted using strain-controlled ARES (TA Instruments, New Castle, DE, USA) and MCR 502 WESP (Anton Paar, Graz, Austria) rheometers using 8 mm plate–plate geometry at 20 °C with a varied gap width according to the gel height. A glass slide containing irradiated keratin gel was fixed on the borders of the lower rheometer plate using double-sided adhesive tape. An adequate amount of 1x assembly buffer was distributed around the sample to prevent the dryness of the gels during the measurement. A solvent trap was placed around the sample to prevent evaporation of the surrounding buffer. As a control, unirradiated keratin samples were prepared and measured under the same conditions. Samples were measured with the following sequence: (i) Short time sweep test for 10 min at a frequency (ω) = 1 Hz and strain (γ) = 2%. (ii) Short frequency sweep from 0.01 to 10 Hz, γ = 2%, 5 data points per decade. (iii) Long frequency sweep from 0.01 to 30 Hz, γ = 2%, 21 data points per decade), (iv) short frequency sweep from 0.01 to 10 Hz, γ = 2%, 5 data points per decade, and (v) transient step rate test.

In the transient step rate test, a steady strain rate of 0.1 s^−1^ was applied to measure the strain-dependent stress in the nonlinear strain regime. The differential shear modulus (K) was determined with a self-written Python script, calculating the gradient of the smoothed stress data divided by the strain step width, rescaled by the linear differential shear modulus. The linear differential shear modulus *K_lin_* is given by the first non-negative value of the smoothed stress data; negative stress values measured for small strains due to technical limitations were excluded.

### 2.4. Keratin Gel Staining and Imaging

For staining, keratin samples were polymerized in an 8-chambered cover glass system (Ibidi GmbH, Gräfelfing, Germany) at room temperature for 1 h. Samples were then prepared as mentioned above and irradiated with 30 kGy, 50 kGy, and 100 kGy. Irradiated keratin gels were fluorescently stained by incubating the gel at the same molar concentration of Atto-488-NHS-ester solution (ATTO-TEC, Siegen, Germany) in phosphate buffer (2 mM sodium phosphate pH 8) for 30–45 min at room temperature. The labelling was based on the coupling of Atto-488-NHS-ester to the free amine (NH2) residues (Lysine or terminus) of keratin after polymerization. After incubation, the dye solution was removed carefully, and the stained gels were washed 3 times with phosphate buffer and imaged immediately. During the whole staining process, samples were protected from the light to avoid dye bleaching. Images were captured using a spinning disc confocal microscope (inverted Axio Observer.Z1/Yokogawa CSU-X1A 5000, Carl Zeiss Microscopy GmbH, Germany), 100× oil immersion objective NA 1.40 with a Hamamatsu camera at an exposure time of 100 ms.

## 3. Results

### 3.1. Irradiation Increases the Elastic Modulus of Keratin Gels

**Figure 1:**
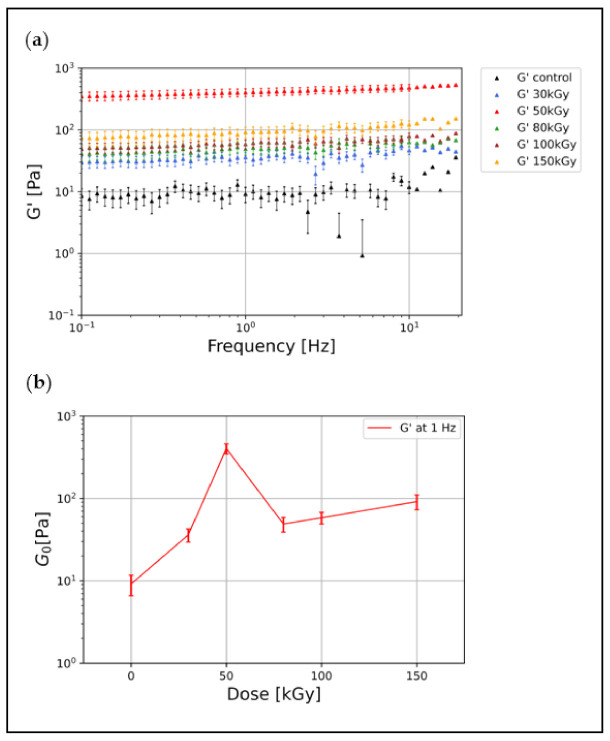
Linear elastic modulus of keratin gels irradiated at different doses in comparison to unirradiated control: (**a**) A pronounced increase in the elastic modulus G′ of all irradiated samples relative to the unirradiated control becomes directly apparent when comparing the frequency sweeps. Testing a broad range of frequencies revealed a very weak frequency dependency of G′ in comparison to other biopolymer networks [9]. (**b**) The plateau modulus G0 (G′ at f = 1 Hz) shows an increase after irradiation until 50 kGy, followed by a remarkable drop starting at 80 kGy. Data points represent the mean of 5 independent measurements and error bars represent the standard deviation from the mean.

Using bulk shear rheology, the linear viscoelastic properties of keratin gels can be quantified by the frequency (ω) dependent complex shear modulus G*(ω) = G′(ω) + iG″(ω), where G′ and G″ are the elastic and viscous moduli, respectively. k8–k18 filament networks at 1.5 mg/mL were irradiated at five different doses (30 kGy, 50 kGy, 80 kGy, 100 kGy, and 150 kGy). We probed the viscoelastic properties of the resulting keratin gels in comparison with unirradiated keratin networks (control). All samples were polymerized at the same concentration and under the same conditions. The viscoelastic properties of the different keratin gels were measured over a broad frequency regime ranging from 0.01 Hz to 30 Hz at a strain of 2%, predetermined to be in the linear viscoelastic region. Our data show that the keratin control samples formed elastic gels with a G′ (at 1 Hz) around 10 Pa (Figure 1a). Covalent crosslinks induced via EBI stabilized the connections between filaments and increased the overall connectivity, i.e., the percolation, in the network leading to an increase in the network’s elastic modulus of all irradiated samples (Figure 1a). This implies that, upon EBI, the keratin networks stiffen due to the chemical crosslinking and increased network connectivity. This increase in G′ is also prominent in the time sweep test, as shown in the Supplementary Information (Figure S1). Compared to the control, irradiation with 30 kGy increased the G′ from 10 Pa to 34 Pa, and irradiation with 50 kGy resulted in a remarkable increase in G′ to 400 Pa.

Interestingly, we found nonmonotonic behavior when irradiating keratin samples with higher doses: 80 kGy, 100 kGy, and 150 kGy. These samples were softened in comparison to the 50 kGy samples showing lower elastic moduli, as seen in Figure 1a. Figure 1b shows this nonmonotonic behavior and how the plateau modulus G_0_ (G′ at 1 Hz) changes with irradiation doses.

It is known that an increasing amount of crosslinking sites in an isotropic network leads to an increase in G′ [8]. Correspondingly, the initial monotonic stiffening of keratin samples with an irradiation dose up to 50 kGy can be explained by increasing crosslinker formation due to increasing OH radical formation. Thus, with an increasing dose, the networks become progressively more percolated. As presented below, we found that these moderate doses of 30 kGy and 50 kGy effectively induced a high degree of percolation in the configuration of isotropic networks. Meanwhile, irradiation doses above 50 kGy induced bundling and thus structural heterogeneities in the network, which caused an overall weakening of the network and thus a drop of G′ [8]. Previously published Monte Carlo simulations supported this possible transition and suggested that bundle-dominated networks contain a larger number of crosslinkers forming bridges between parallel filaments within a single bundle, and fewer contribute to overall network percolation as the degree of bundling becomes more pronounced [44]. In our case, bundle formation leads to local anisotropies and fewer filaments contributing to the percolated network.

Previous rheological characterizations of k8–k18 in vitro networks have shown that there are inherent attractive interactions between filaments within the network [9,17]. These interactions play a key role in the arising network mechanics and easily overwrite the influence of other contributions, which were previously thought to be determining factors [9,17,45]. For both physically connected (control) and chemically crosslinked (irradiated) keratin gels, the motion of filaments within the networks will be highly restricted or even completely stalled in irradiated gels, which is mirrored in the weak frequency dependency of G′, as seen in Figure 1a.

One characteristic feature of keratin networks is the high elasticity, which is directly shown in the small value of the loss factor tan (ϕ) = G″/G′ (tan (ϕ) at 1 Hz = 0.161). After irradiation, we have observed that the increase in G′ was also accompanied by an increase in the viscous modulus G″, which results in an almost constant loss factor (Figure S2). A similar behavior of the increased viscous modulus was also reported for irradiated collagen and gelatin gels, which was attributed to random crosslinking sites introduced through irradiation and the potential formation of dangling ends [29,46]. In a prior study, a hydrogel of hair and wool keratins blended with synthetic polymers have been prepared by EBI, displaying increased mechanical strength with increasing irradiation dose, followed by saturation [41].

### 3.2. Networks Architectures

**Figure 2:**
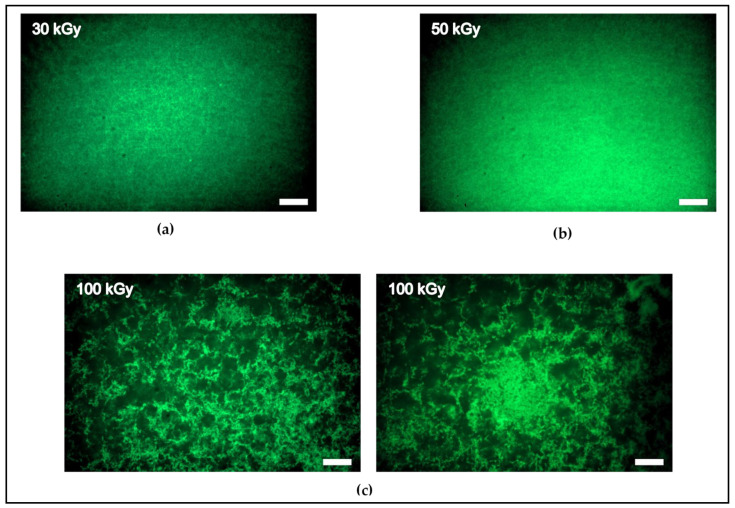
Spinning disc confocal microscopy images of keratin gels showing the impact of different irradiation doses on keratin architecture. Irradiation with 30 kGy and 50 kGy created highly percolated isotropic networks (**a**,**b**). Irradiation with 100 kGy induced the formation of thick bundles and clusters rendering the network heterogeneous and weakly connected (**c**). Scale bar: 10 µm.

To investigate the impact of crosslinking on the structure of keratin, we used spinning disc confocal microscopy to visualize the architecture of keratin irradiated with 30 kGy and 50 kGy, and, from the higher doses, we selected the 100 kGy. Under our assembly conditions and without irradiation, keratin does not tend to form bundles or clusters but rather forms an entangled network with homogenous architecture [12]. After irradiation, the images revealed that the keratin networks still display an isotropic configuration after irradiation with 30 kGy and 50 kGy. Thus, these doses induce stiffening of the network via crosslinking and increasing percolation in these isotropic networks (Figure 2a,b). Moreover, there is no observed difference in the architecture of the samples of both doses. In stark contrast, irradiation with 100 kGy, i.e., a higher degree of crosslinking, significantly impacted the architecture, producing thick bundles and clusters that weaken the percolation of the network (Figure 2c).

This is in good agreement with the rheological behavior, and the significant structural difference between the moderate and high doses can explain the nonmonotonic behavior of the linear elastic moduli. Fully connected isotropic networks of 30 kGy and 50 kGy displayed an increase in the elastic modulus until reaching 50 kGy, while the emergence of heterogeneity in the 100 kGy reduced the elastic modulus by almost one order of magnitude.

### 3.3. Nonlinear Rheology

#### Irradiation Suppresses the Nonlinear Strain Stiffening of Keratin

Within the linear regime, only small deformations are tested. However, in reality, cells often experience larger strains that exceed their linear regime. Therefore, unlike most synthetic polymers, many biopolymers respond to deformations nonlinearly by displaying an increasing stiffness, which is called strain stiffening or strain hardening, which typically reflects individual filament behavior [4,19,47].

**Figure 3:**
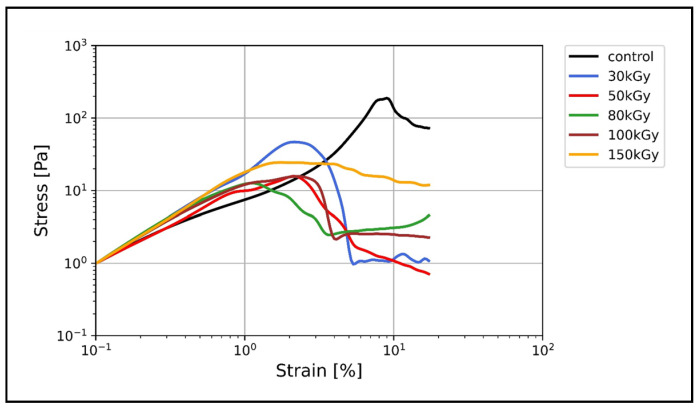
Stress–strain relationship of all keratin networks. With increasing strain, keratin control displayed the highest stress response compared to irradiated samples. Keratin control showed the yield stress (maximum stress) at a higher strain rate relative to irradiated keratins. The change (increase) of the slope of the stress–strain relation (reflecting the strain stiffening) is clearly observed only in the control, which implies the limited ability of irradiated keratin networks to stiffen in response to the applied steady strain.

k8–k18 in particular displays profound nonlinear strain stiffening without any kind of crosslinking [16,17]. Figure 3 shows the stress–strain relation of all keratin networks. In the unirradiated keratin, three distinct regimes can be identified: a regime with relatively low stiffness, i.e., the ratio of stress to stain, a transition regime (the onset of nonlinear stiffness), and a high stiffness regime. The transition regime where the stress starts to increase nonlinearly with an increasing strain rate is reflected by the obvious slope change in the stress–strain relation. Interestingly, in contrast to unirradiated keratin networks, this slope change is not observed in the stress–strain relationship of irradiated keratin networks. Only for the low irradiation dose of 30 kGy could a minor slope change be conceivable (Figure 3). Furthermore, the unirradiated keratin samples displayed the highest stress response with σmax = 188 Pa at 9% strain. The 30 kGy-treated samples displayed lower σmax of 46 Pa at 2.3% strain, while the other irradiated samples showed much lower stress responses (12–20 Pa) at comparable strain rates (1–2%).

In order to quantify the strain stiffening, we calculated the differential shear modulus $K=\frac{d\sigma }{d\gamma }$ as the local derivative of stress σ over strain γ. In the linear regime, the differential shear modulus is constant, which means that the stress response in this regime is independent of the applied strain. K increases when the network displays strain stiffening and remains almost constant before it quickly drops when the network strain softens before it breaks.

**Figure 4:**
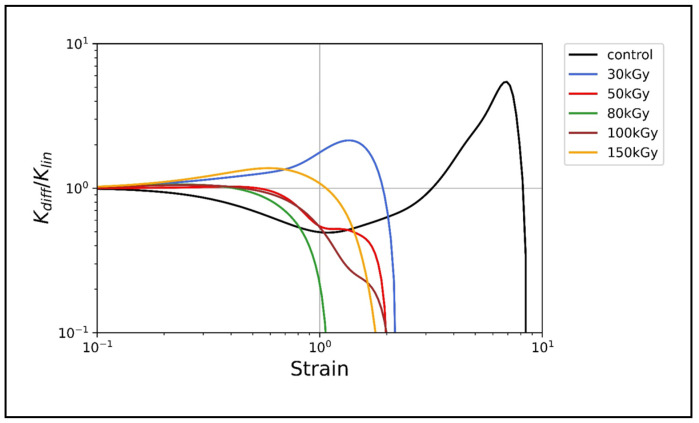
Differential shear modulus K=dσ/dγ rescaled by its value in the linear regime *K_lin_* as a function of strain. Keratin control showed strain stiffening, as indicated by the increase in *K* with an increasing strain rate, stiffening by a factor of 5. Irradiated keratins showed a weak (at 30 kGy) to no (at other doses) strain stiffening behavior, but rather a strain softening.

The data presented in Figure 4 show that only the keratin control shows a strong strain stiffening, as evidenced by the maximum value of $\frac{K}{Klin}\;$= 5.5 at a higher strain rate in comparison to irradiated samples. Networks irradiated at 30 kGy showed a relatively weak stiffening behavior with a maximum $\frac{K}{Klin}=$ 2.14, while at higher doses the networks did not show any stiffening behavior, as indicated by the quick drop of $\frac{K}{Klin}$.

## 4. Discussion

Intracellular keratins undergo different types of crosslinking with divergent impacts on their structure and mechanics and thereby functionalities. Here, we showed that the EBI is a convenient tool for k8–k18 crosslinking and investigated the rheology and architecture of the crosslinked keratin structures. Due to the formation of covalent crosslinks after irradiation, the network connectivity has increased, increasing the linear elastic modulus G′ of keratin in all doses tested (30 kGy, 50 kGy, 80 kGy, 100 kGy, and 150 kGy) (Figure 1a). As the irradiation dose increases, the crosslinking density increases, and thus the network becomes progressively more percolated, displaying higher G′. We found an initial increase in G′ with irradiation up to 50 kGy, displaying the maximum G′ value (from 10 Pa to 400 Pa). In a prior study, preparation of hair and wool keratins hydrogels via EBI was possible only after they were blended with either a synthetic polymer (poly–vinyl alcohol) with irradiation at a dose higher than 90 kGy, or with the addition of poly–ethylene imine to the blend with irradiation at 10 kGy [41]. This keratin-synthetic polymers’ hydrogel displayed increased gel mechanical strength with increasing irradiation dose. Here, however, we showed that a considerably lower dose of 30 kGy was sufficient to induce crosslinking of pure k8–k18 networks without any additives, yielding pure keratin-based hydrogel with a higher elastic modulus than the unirradiated one. As the confocal microscopy imaging revealed, both the 30 kGy and 50 kGy-treated samples possess completely isotropic and obviously highly percolated architectures (Figure 2a,b). However, a remarkable drop in the elastic moduli in comparison to the 50 kGy accompanied irradiation with higher doses. This is attributed to the emergence of bundling and anisotropies as clearly seen in the architecture of 100 kGy-treated keratins (Figure 2c). All keratin networks treated with doses higher than 50 kGy displayed comparable elastic moduli, which implies a saturation or a maximal percolation with no further effect of crosslinking on the mechanical properties of the networks. Similar behavior has been reported earlier for keratin, gelatin, and collagen gels after irradiation [29,41,46].

One hallmark of keratins is their inherent ability to significantly stiffen under increasing strain and thereby protect cells and tissue under high deformation forces [12,20,21,48]. This behavior, in general, has attracted theoretical attention to address the origin of strain stiffening in biopolymer networks. The prevalent view attributes the network stiffening primarily to the entropic, longitudinal stiffening of the constituting individual filaments under the assumption that the network deforms in an affine manner (each filament follows the overall deformation) [19,49,50]. Alternative theories adopted the idea that the overall stiffening response could also originate from the network itself, rather than from its constituents assuming nonaffine network rearrangements [51,52,53]. The strain stiffening of keratin, and IFs in general, was attributed to the large extensibility of keratin filaments since their hierarchical structure enables the partial unfolding of subunits without filament breakage [47,54]. Our results revealed that, after irradiation, crosslinked keratins lose their strain stiffening ability, displaying unusual strain softening, while the entangled networks of unirradiated keratins significantly stiffen (Figure 4). As indicated by the increase of the differential shear modulus K, only 30 kGy-treated keratins exhibited a weak strain stiffening, which, however, is still lower than the normal stiffening of keratin control. It was previously shown that, for keratin bundles, the coupling between filaments in thick bundles is weaker than that in thin bundles [55]—this weak coupling allows more filament sliding under strain and thus does not support strain stiffening. Here, however, the 50 kGy isotropic bundles-free networks displayed strain softening similar to the bundles-containing networks of 100 kGy. Therefore, it seems that neither the network percolation nor the bundling is the main effector for the nonlinear response but that it is most likely EBI-related chemical changes. In previous work, electron beam-treated keratin and elastin–collagen gels exhibited a reduction in the α-helical content with a concomitant increase in β-sheet and random coil, respectively [30,34]. This unfolding was conceptualized before as the origin of strain stiffening; furthermore, studies on hard keratin, keratin-like bundles, and vimentin (another IF protein) have confirmed the loss of the α-helical structure and the creation of an extended β-sheet upon stretching [56,57,58]. 

Taken together, we hypothesize that the α-helix to β-sheet unfolding already has occurred in k8–k18 after irradiation, and this rendered the filaments inextensible and thereby suppressed the strain stiffening. The weak strain stiffening observed only in the 30 kGy-treated samples supports our hypothesis, as the lowest dose may still allow partial extension of filaments and thereby limit strain stiffening capability. In comparison, filaments within the unirradiated keratin networks with the characteristic α-helical motif are able to stretch to their maximum length to accommodate the large strain, showing the observed strain stiffening. 

The loss of the mechanical resilience of cells in keratin-related diseases was mainly attributed to keratin mutations that disrupt filament formation [59]. However, we have demonstrated here the possibility of the loss of strain stiffening in properly assembled keratin as a consequence of covalent crosslinking. A potential crosslinking reaction through EBI is via covalent binding between lysine and glutamine amino acids. This covalent binding was previously determined in heated wool keratin and irradiated collagen gels [60,61]. Interestingly, a similar reaction catalyzed by transglutaminase (TG) enzymes takes place in the cell as one of lysine post-translational modifications [62]. Both epidermal and epithelial keratins undergo such modifications, albeit with divergent consequences for both keratin types [63]. In epidermal keratins, this crosslinking attaches keratin to the cell envelope and enhances the maintenance of the skin barrier function [64,65]. In contrast, in k8, this crosslinking is associated with the formation of hepatocytes, inclusions called Mallory–Denk bodies (MDB), which are k8–k18-containing aggregates, and their presence serves as a poor prognostic marker in liver diseases [66]. Importantly, as a result of this crosslinking, k8 acquires increased β sheet structure at the expense of reduced α helices [67]. 

To our knowledge, a simple method for the in vivo detection of protein crosslinking and resultant structural and mechanical alteration is lacking. The feasibility of EBI to achieve crosslinking will facilitate further investigations of the impact of crosslinking on molecular structure, mechanics, and other protein properties. 

## 5. Conclusions

We have shown the feasibly of EBI to covalently crosslink k8–k18 filament networks and investigated the rheology and architecture of the resultant crosslinked keratin gels. Irradiated keratins possess a higher linear elastic modulus than the unirradiated ones at all doses tested; the elastic modulus initially increased with an increasing irradiation dose until 50 kGy, and subsequently decreased with higher doses. This was clearly correlated with the degree of network percolation, as the microscopy images revealed. Under increasing strain, the crosslinking has prevented keratin filaments from extending, most likely due to conformational changes after irradiation, which suppressed the strain stiffening ability in irradiated keratins. Our results provide new insights into the contribution of covalent crosslinking to the loss of mechanical resilience in keratin-related diseases. From another perspective, keratins possess unique material properties, including powerful self-assembly, mechanical resilience, and quick recovery after breakage, in addition to the capability to “switch off” the strain stiffening, as we have shown here. As these properties can be better understood and controlled, this can be exploited to create innovative keratin-based biomaterials for specific applications in cell culture and biomedicine.

## Data Availability

Not applicable.

## Supplementary Materials

The following supporting information can be downloaded at: https://www.mdpi.com/article/10.3390/polym14030614/s1, Figure S1: Time sweep test; Figure S2: Influence of irradiation on loss factor.
