# Supplementary material for: From Strain Stiffening to Softening—Rheological Characterization of Keratins 8 and 18 Networks Crosslinked via Electron Irradiation
Source: Polymers (Basel). 2022 Feb 4;14(3):614. doi: 10.3390/polym14030614 (PMC8838340; doi:10.3390/polym14030614)
Supplement: Supplementary file 1 [file polymers-14-00614-s001.zip › polymers-1561264-supplementary.pdf]

From strain stiffening to softening - Rheological characterization of keratin networks crosslinked via electron irradiation

Iman Elbalasy [1,2,*], Nils Wilharm [3,4], Erik Herchenhahn [1], Robert Konieczny [3], Stefan G. Mayr [3,4] and Jörg Schnauß [1,5,6,*]

1 Peter-Debye Institute for Soft Matter Physics, Leipzig University, Linnéstraße 5, 04103 Leipzig, Germany; iman.elbalasy@uni-leipzig.de; eh12veli@studserv.uni-leipzig.de; joerg.schnauss@uni-leipzig.de
2 Faculty of Science, Cairo University, Giza, Egypt
3 Leibniz-Institut für Oberflächenmodifizierung e.V. (IOM), Permoserstr. 15, 04318, Leipzig, Germany; nils.wilharm@iom-leipzig.de; robert.konieczny@iom-leipzig.de; stefan.mayr@iom-leipzig.de
4 Division of Surface Physics, Department of Physics and Earth Sciences, Leipzig University, Linnéstraße 5, 04103, Leipzig, Germany.
5 Fraunhofer Institute for Cell Therapy and Immunology, 04103 Leipzig, Germany.
6 Unconventional Computing Lab, Department of Computer Science and Creative Technologies, UWE, Bristol BS16 1QY, UK.
* Correspondence: iman.elbalasy@uni-leipzig.de (I.E.); joerg.schnauss@uni-leipzig.de (J.S.).

Time sweep test

The time sweep test captures the time evolution of the elasticity during polymerization. In this test, the amplitude of the oscillatory strain was fixed at $\gamma = 2\%$, predetermined to be in the linear viscoelastic region, and deformation frequency at ω = 1 Hz. Since all keratin samples have been polymerized prior to the rheological measurements, the elastic moduli of the networks remain unchanged over time (figure S1).

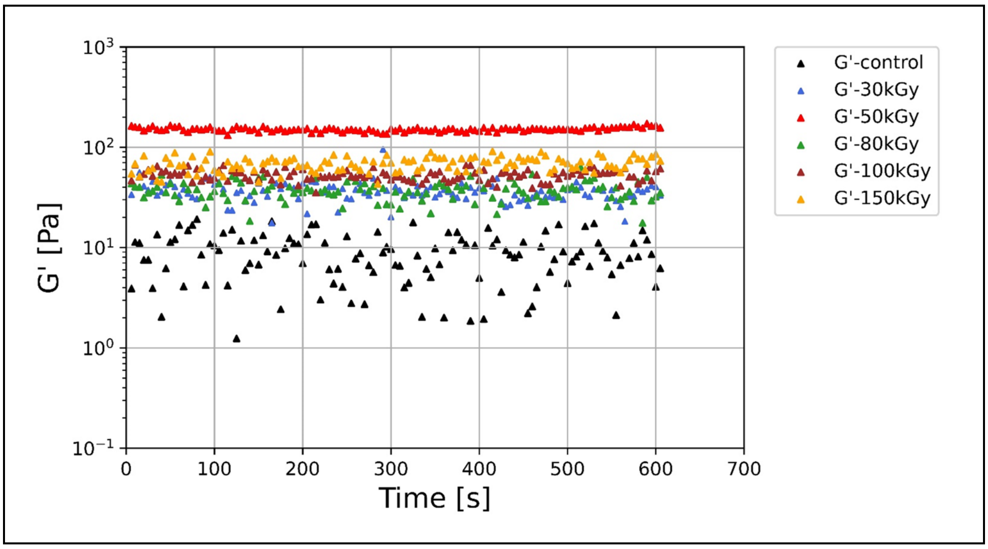

Figure S1. The time sweep test shows a very weak to no time dependency of the elastic modulus (G') for both irradiated and control keratin gels.

Influence of irradiation on loss factor

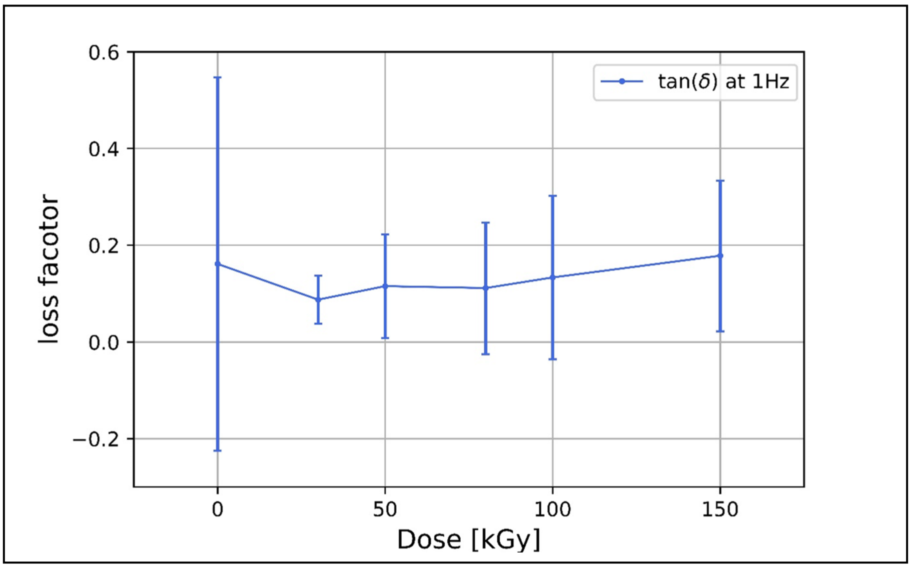

Figure S2. Mean values of the loss factor tan (ϕ) at f = 1 Hz of keratin gels at different doses show only minor variation in comparison to the control.
